# Thoracoscopic traction technique in long gap esophageal atresia: entering a new era

**DOI:** 10.1007/s00464-015-4091-3

**Published:** 2015-02-11

**Authors:** David C. van der Zee, Gabriele Gallo, Stefaan H. A. Tytgat

**Affiliations:** 1 Department of Pediatric Surgery KE.04.140.5, Wilhelmina Children’s Hospital, University Medical Center Utrecht, P.O. Box 85090, 3508 AB Utrecht, The Netherlands; 2 Department of Pediatric Surgery, University Medical Center, Groningen, The Netherlands

**Keywords:** Esophageal atresia, Long gap, Thoracoscopy, Traction technique

## Abstract

**Objective:**

To describe the evolution from delayed management of long gap esophageal atresia to thoracoscopic treatment directly after birth without the placement of a gastrostomy.

**Background:**

Long gap esophageal atresia remains a challenge for pediatric surgeons. Over the years, several techniques have been described to deal with the problem of the distance between the proximal and distal esophagus. More recently, a traction technique has been advocated. With the advent of minimal invasive surgery, the thoracoscopic elongation technique has been developed.

**Methods:**

Retrospective description of a single-center experience with the thoracoscopic treatment of patients with long gap esophageal atresia over a 7-year period.

**Results:**

Between 2007 and May 2014, 10 children with long gap esophageal atresia were treated by thoracoscopic elongation technique. In two children, the procedure failed. Eight children successfully underwent thoracoscopic traction with delayed primary anastomosis. Initially, all patients had a gastrostomy. During the course, the technique evolved into delayed primary anastomosis directly after birth without the use of a gastrostomy.

**Conclusion:**

Thoracoscopic elongation technique in long gap esophageal atresia not only is feasible, but can nowadays also be performed directly after birth without the use of a gastrostomy. With this development, we have entered a new era in the management of long gap esophageal atresia.

Long gap esophageal atresia remains a challenge for pediatric surgeons. Over the years, several techniques have been described to tack the problem of the distance between the proximal and distal esophagus. The incidence of long gap esophageal atresia is so low that it is difficult for individual centers to gain large experience and most series published have anecdotal data.

A more recent developed technique is the open traction technique, first described by Foker [1], in which the two ends of esophagus are pulled toward each other by external traction over time to ultimately be anastomosed. The outcome is variable, and achievement of feeding is not undivided favorable [2, 3]. With the advent of minimal invasive surgery and the increasing experience in the treatment of type C esophageal atresia, the thoracoscopic elongation technique became feasible. After a first description of the technique [4], we now describe our 7-year experience with the thoracoscopic traction technique and the development toward a procedure almost similar to the standard type C esophageal atresia.

## Materials and methods

### Evolution of technique

Initially, we started with performing a (laparoscopic) gastrostomy upon the diagnosis of long gap esophageal atresia together with a Replogle suction tube in the proximal esophagus. Along the course, as we started the traction directly after birth, we no longer performed the gastrostomy, but only did a laparoscopic gastropexy against the anterior abdominal wall to prevent the stomach from migrating up into the thorax. We principally try to avoid an esophagostomy in the neck, because it will be more difficult to bring the esophagus back down into the thorax at a later stage, reducing the available techniques usually to a gastric pull-up or colon interposition.

Each procedure is started with a rigid tracheobronchoscopy as almost half of our patients turned out to have a proximal fistula. Depending on the level of the proximal fistula, this is managed either thoracoscopically or through the neck.

For the traction technique, the patient is positioned in a ¾ left lateral position at the left side of the table, as we would do for the routine thoracoscopic anastomosis in type C esophageal atresia. A first 5-mm trocar is placed 1 cm anterior and below the tip of the scapula by incision in the skin and blunt perforation of the muscularis and pleura, respectively. In smaller children under the weight of 2,000 g, we increasingly use a 3-mm trocar for the optic. Thereafter, two 3-mm trocars are placed under direct vision in a triangle around the endoscope.

All patients nowadays are operated upon under the surveillance of near-infrared spectrometry (NIRS) and a-EEG to monitor the brain oxygenation and activity, respectively.

**Fig. 1:**
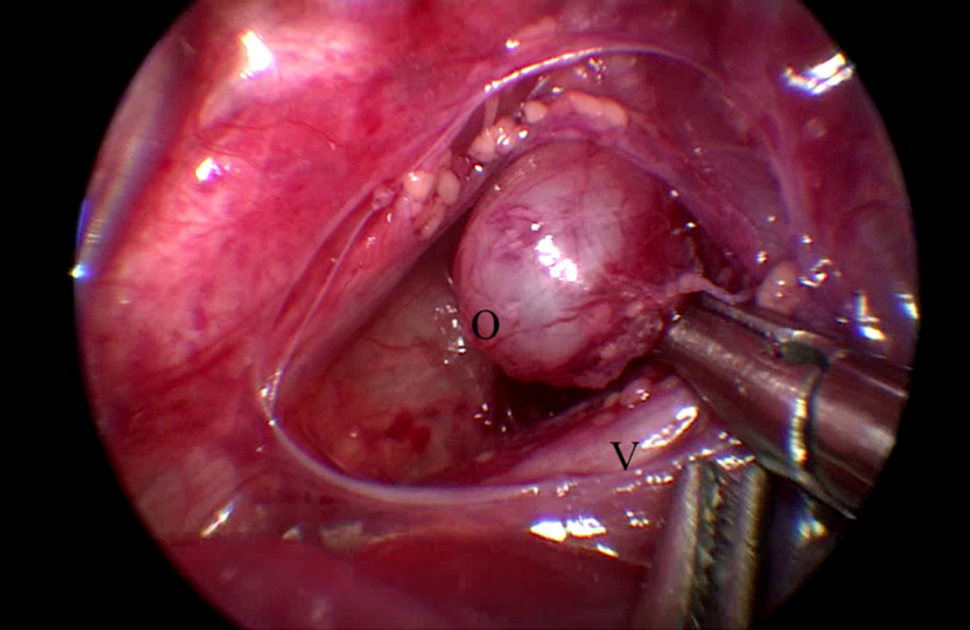
Mobilization of proximal esophagus. O = proximal esophagus, V = trachea with onlying vagal nerve

After insufflation with CO_2_, at 3–5 mm Hg and a flow of 1 l/min, and adjustment of the ventilation by the anesthesiologist, it is started with mobilization of the proximal esophagus to a maximal extent in the thoracic aperture (Fig. 1). If a proximal fistula is present, this is closed at the same instance.

**Fig. 2:**
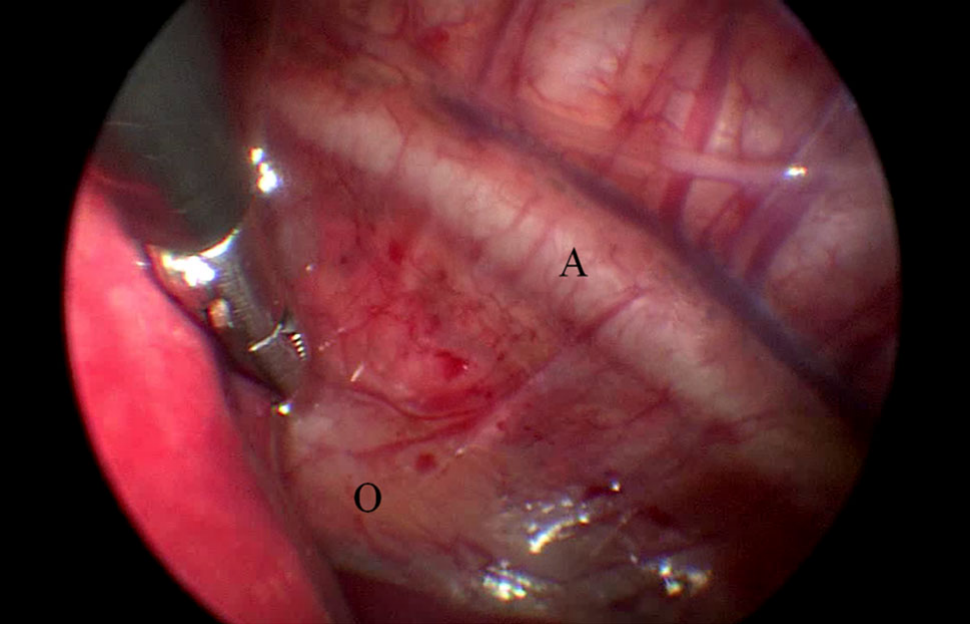
Mobilization of distal esophagus out of hiatus. O = distal esophagus coming through the esophageal hiatus, A = aorta

**Fig. 3:**
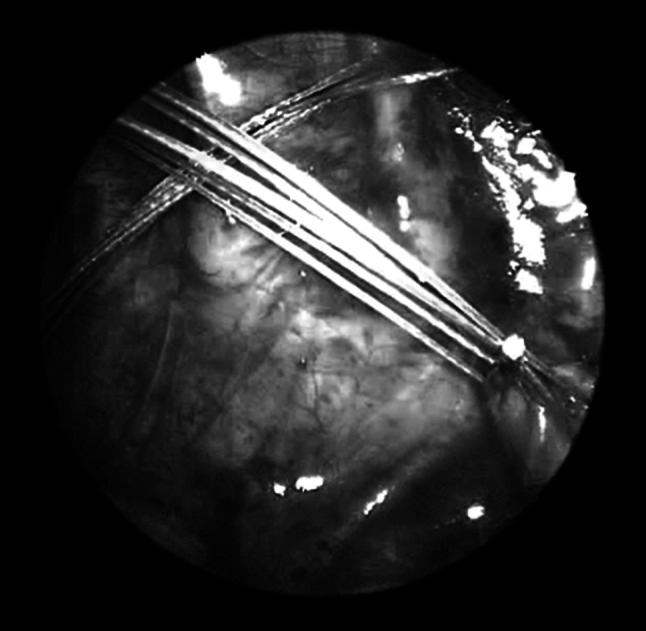
Traction sutures with a clip close to the esophageal pouches

**Fig. 4:**
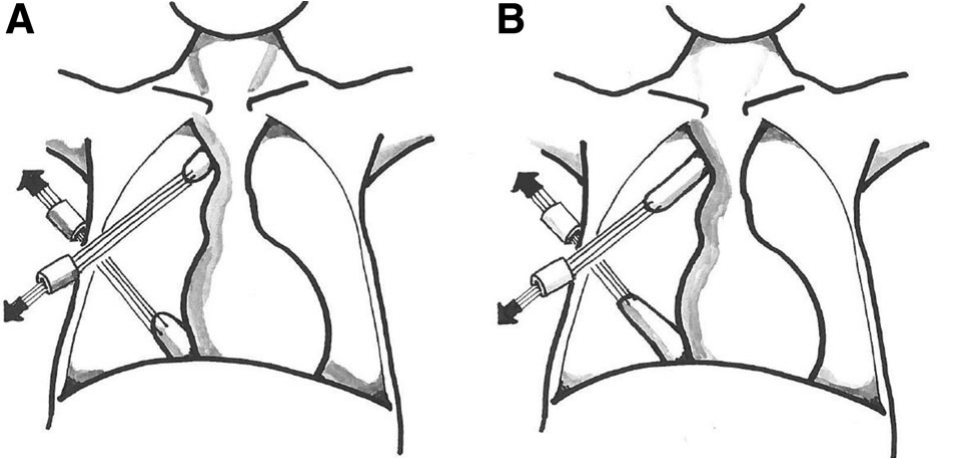
Diagram of traction technique. **A** Distance at start of traction. **B** Elongation of the two pouches over the days of traction

Thereafter, the distal esophagus is determined (Fig. 2) and mobilized out of the esophageal hiatus. Frequently, the hiatus has to be opened in order to retrieve the distal esophagus. The esophagus is mobilized as much as possible up to the fundus of the stomach. Principally, all patients will need an antireflux procedure at a later stage. Traction sutures Vicryl 4 × 0 (Ethicon, Johnson & Johnson, Amersfoort, NL) are introduced with the use of an Endoclose^®^ (Covidien, Zaltbommel, NL), and bites of the esophagus are taken at four corners. Pledgets have not been used. Again with the Endoclose^®^, the sutures are crosswise withdrawn from the thorax and through a small piece of silicone tubing held with a mini-mosquito under traction. The same procedure is carried out on the other side. Close to both ends of the esophagus, a clip is applied to the sutures (Fig. 3) to be able to determine the approximation over the coming days by thorax radiograms. Under direct vision, the traction is tested and the distance to be covered is determined. The procedure is then terminated. The 5-mm defect is closed with a Vicryl 5 × 0 muscular and subcutaneous suture, and all skin defects are approximated with Steristrips^®^ (3 M, Zoeterwoude, NL). During the traction period, the patients remain intubated and sedated, but there is no need to be paralyzed. A diagram displays the principal of the procedure (Fig. 4).

Nowadays, the patient, that is referred without gastrostomy as well as the patient primarily born in our center, is then turned in a supine position, and a 5-mm trocar is introduced through the umbilicus by open technique. One or two additional 3-mm trocars are placed under direct vision. The (micro-) stomach is located, and the best spot is determined to perform a gastropexy against the ventral abdominal wall with two Ethibond 4 × 0 sutures (Ethicon, Johnson & Johnson, Amersfoort, NL) to prevent the stomach from migrating into the thorax.

**Fig. 5:**
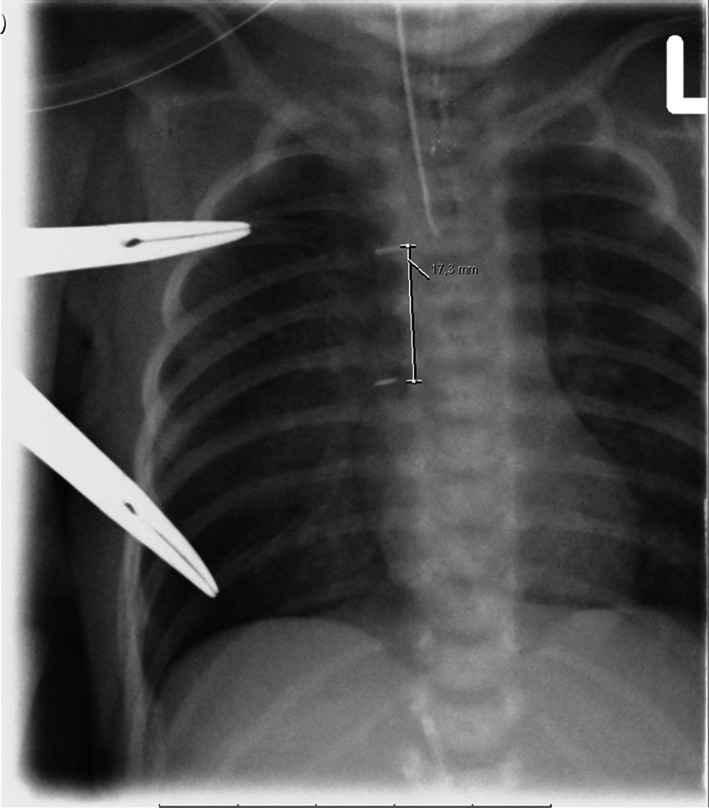
X-thorax after application of traction sutures. There is still a distance of 17.3 mm

A postoperative X-Thorax is made to determine the length of the defect (Fig. 5), and the approximation is followed by daily radiograms. The traction sutures are checked twice daily, but unless there is a lot of mobility, the mosquitos are not adjusted and no additional traction is exerted, as too much traction will lead to disruption of the sutures. This detail is crucial in our opinion, because since having this restraining protocol, no more suture disruptions have occurred.

Usually, after 3–4 days, there is no more progression, due to adhesion formation between the esophagus and the adjacent lung. The child is then taken back into the operating theater, and thoracoscopic adhesiolysis is carried through by carefully sweeping loose the adhesions between esophagus and lung. Usually, there is still a too large gap between the two ends to safely perform a primary anastomosis. If necessary, the sutures can be led out at a higher level, and traction is installed again.

**Fig. 6:**
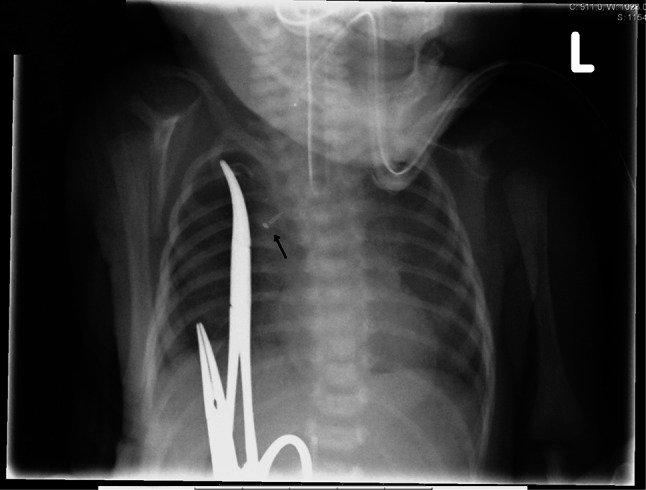
X-thorax after 5 days. The clips of the proximal and distal pouch have reached each other (*arrow*)

**Fig. 7:**
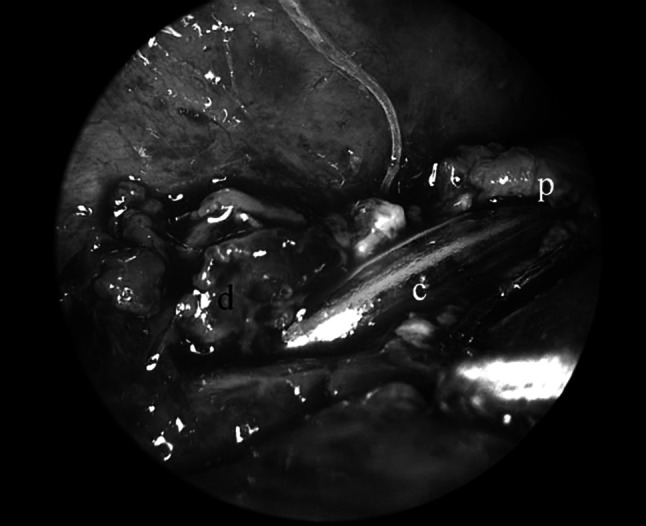
Advancing nasogastric tube after anastomosis of posterior wall. p = proximal esophagus, d = distal esophagus, c = feeding tube

In general, after a total of 4–6 days, when the clips have approximated sufficiently (Fig. 6), the patient can be taken back to theater for the delayed primary anastomosis. After mobilization of the two ends, two or sometimes three traction sutures can be applied at the corners and posterior wall of the two pouches, before opening the proximal and distal esophagus, and the two ends can be advanced by the sliding technique. One or two additional sutures can be laid on the posterior wall before a 6–8F gastric feeding tube is advanced into the distal esophagus and stomach (Fig. 7).

Sometimes the mucosa in the distal esophagus has not advanced as much as the muscularis, and the distal esophagus has to be incised further to identify and open the mucosa. This can hamper making a solid anastomosis.

Principally, a drain is only left behind if there is doubt that the anastomosis is 100 % watertight.

A contrast swallow study is performed at day 5. When there is no leakage, oral feeds can be started. In patients that have a micro-stomach, this can be difficult, and in those cases, often it has to be started with continuous drip feeding giving the stomach time to adjust and grow.

In case there is no advancement or when complications occur, such as perforation, the technique is abandoned, and management is switched to alternative procedures like jejunal interposition or gastric pull-up, in case the proximal esophagus is too high up in the thorax or the neck.

Principally, all children will need a laparoscopic fundoplication after 4–6 weeks.

The study was approved by the hospital medical ethical committee.

## Results

Between 2007 and May 2014, 10 children were either admitted or transferred to our department for treatment of their long gap esophageal atresia. Gestational age varied from 30 4/7–40 1/7 weeks (*M* = 34 4/7). Weight at time of birth varied from 1,395 to 3,850 g (*M* = 2,330 g). Age at time of operation varied from 2 days to 6 months (Table 1). In four patients, a proximal fistula was detected during preoperative tracheoscopy. In two cases, the fistula could be closed thoracoscopically, the two others were too high and were dealt with through the neck.

**Table 1:** Demographics of patients with long gap esophageal atresia

Patients	2007–May 2014
No.	10
Boys	6
Girls	4
Gestational age	30 4/7–40 1/7 weeks (mean 34 4/7)
Birth weight	1,395–3,800 g (mean 2,330 g)
Associated congenital anomalies	AVSD 1
	ARM 2

*AVSD* atrium-ventricular septum defect, *ARM* anorectal malformation

Initially, the patients either received a gastrostomy or were referred with a gastrostomy and a Replogle tube in the proximal esophagus. As of the fifth case, we no longer performed a gastrostomy, but kept the patient on parenteral nutrition during the elongation period. The first time we performed the procedure without gastrostomy, we encountered that after 2 days, the two pouches could be easily anastomosed, but that the stomach had migrated partially into the thorax. We thereafter prophylactically performed an anterior gastropexy against the anterior abdominal wall to prevent the stomach from going up into the thorax. The first time, however, we experienced that the Vicryl 5 × 0 suture we used had partially dissolved when performing the laparoscopic antireflux operation 6 weeks later. We since then use Ethibond 4 × 0 to fix the stomach against the anterior abdominal wall.

In two cases in the early experience, the traction sutures have torn out during the traction procedure and had to be replaced. It was therefore decided not to apply additional traction on the sutures during the elongation, unless there was evidently no tension on the sutures any longer, in order to prevent disruption by pulling too hard. Since restraining the protocol, no more suture ruptures have occurred. In one 1,710-g child, after 5 days, the end of the pouches seemed partially frayed by the past traction, still leaving approximately 1-cm bridge to gap during anastomosis. The child, however, recovered well with no leakage at the contrast study after 5 days. In four additional cases, there was no further advancement after 3 days, and we had to go back to perform adhesiolysis to facilitate further traction. In one of these children, the clip of the distal pouch had reached the thoracic wall and during this procedure was replaced two ribs higher (Table 2).

**Table 2:** Distance between proximal and distal esophagus after maximal traction

Patient	Distance in no. vertebrae after maximal traction	Distance in mm between clips after maximal traction
1	3	21.6
2	5	35.8
3	4	29.8
4	3	23.9
5	2	11.9
6	3.5	21.5
7	3	19.1
8	3	19.6
9	3	17.0
10	3.5	24.5

In one child, there was no more advancement after 5 days, and we had to undo the anterior gastropexy in order to gain more length and make the primary delayed anastomosis. At this time, this did not have any negative effect on the abdominal position of the stomach, as could be determined during the antireflux procedure 6 weeks later.

In two patients, the elongation procedure failed. The first time was a patient, where we only minimally dissected the two pouches before applying the traction sutures, reasoning that if induced growth, as was suggested by Foker, was the crucial factor in elongation, then minimal dissection would suffice and reduce the risk of compromised perfusion. However, no gain of length was achieved, and eventually the sutures were torn out of the distal esophagus, and there was an open connection with the lumen. The procedure was therefore abandoned, and the patient underwent a jejunal interposition. In the second patient, there was an accidental perforation of the proximal pouch with the Replogle tube by the anesthesiologist during dissection. The perforation was closed, and traction sutures could be applied. In the days thereafter, the two pouches approached satisfactorily, until after 3 days during changing endotracheal tube plasters, the Replogle tube was accidentally advanced, and again caused a perforation of the proximal esophagus. On re-exploration, there was contamination of the mediastinum, and the distance was still too large to be bridged. As the upper pouch was high up in the thorax aperture, it was decided to perform a gastric pull-up.

Postoperatively, in two children, there was some minor leakage for which a drain was placed for 3 days. The others could start drinking 5 days postoperatively. The children could be discharged 14–20 days postoperatively, meaning that the last four patients that were treated without gastrostomy could be discharged at the age of 16–21 days.

In the follow-up, all but one children had gastroesophageal reflux requiring dilatation and underwent a laparoscopic antireflux procedure after 4–6 weeks. Three children additionally needed balloon dilatation thereafter, but are now free of symptoms. Two children suffered from life-threatening events due to severe tracheomalacia and underwent a thoracoscopic aortopexy.

All children grow and eat according to their age.

## Discussion

Long gap esophageal atresia has always been a challenge for the pediatric surgeon. In the past, initially all patients were given a gastrostomy for feeding. During the follow-up after 2–6 months, a contrast study could be performed to determine the distance between proximal and distal end of the esophagus. It was then decided how to approach the defect. In some patients, a delayed primary anastomosis could be attempted, and in others, it was chosen for esophageal replacement by gastric pull-up, jejunal or colon interposition [5–7].

There has been ongoing discussion if the native esophagus is not the best option for restoring the continuity. In 1997, Foker described his external traction technique. He hypothesized that the native esophagus would grow under stimulation of traction [8]. If that would be the case, this growth would be exceedingly fast. In our second patient, we only minimally dissected both ends of the esophagus, in order to let growth take place without compromising the circulation during extensive dissection. However, there was only minimal stretching without any progress as suggested by Foker. We therefore doubt that growth will be of any important influence in the advancement of both ends of esophagus. Length will primarily be gained by traction and distraction. In all the other patients that underwent the thoracoscopic elongation technique, sufficient length was achieved within 4–6 days of traction. In our ninth patient, elongation did not extend further than 5 days. Prolonged traction did not lead to further gain of length, and during the procedure for restoring continuity, the anterior gastropexy was released in order to gain more length. Although our experience is still limited, we do not believe that traction longer than approximately 10 days will be adding anything in the gaining of length. What is important is the fact that the tissues of esophagus and lungs will adhere in due time. In open surgery, all kinds of silicone sheeting are used to avoid adhesion formation. In the thoracoscopic approach, this is not feasible, and keeping the procedure as simple as possible, after 3–4 days when no more progression is seen, renewed thoracoscopy is performed to carefully release the adhesions and ascertain that the traction sutures are still effectively in place, as was the case in three patients.

Another issue is what kind of sutures should be used and how deep the bites should be taken. Surely one can take superficial 6 × 0 sutures, using pledgets to protect the tissue from tearing, but this will carry the risk that the underlying mucosa will not advance likewise. Even when using Vicryl 4 × 0 sutures, taking good bites, in two cases we encountered retraction of the mucosa in the distal esophagus. In one case, we could introduce a dilator through the gastrostomy and advance the mucosa for suturing, and in the other, we had to incise the distal esophagus over 1 cm to retrieve the mucosa.

In our experience, it has particularly been the distal esophagus that could be elongated. The proximal end extended either only slightly or none at all. Taking into account the fact that the fetus has been trying to swallow its amniotic fluid throughout pregnancy, it seems logical that the proximal esophagus has already been stretched maximally and that not much gain is to be expected, apart from releasing a proximal fistula. We therefore have some reservation as to the Kimura technique [9]. Mobilizing the proximal esophagus into the neck and trying to elongate it is an extensive procedure, not only in time, but also bringing it back into the thorax, not to speak of the discomfort for the patient. Externalizing the esophagus into the neck will make a secondary intrathoracic anastomosis, with either the distal esophagus or an interpositioned jejunum, more difficult. Nowadays, continuous suction with a Replogle tube is a well-accepted method [10].

As the long gap esophageal atresia repair is complicated, usually time is bought by creating a gastrostomy for enteral feeding and letting the child grow, before an attempt is made to perform a delayed primary repair or the interposition of either stomach, jejunum or colon. Before starting on the thoracoscopic elongation technique, extensive experience was achieved with the thoracoscopic correction of type C esophageal atresia [11]. Dealing with these cases, we also encountered patients where the distance between the proximal and distal pouch extended over several centimeters. With the use of sliding knot suture technique, we managed to approximate these esophageal ends to make a sufficient anastomosis. All these procedures were carried out in neonates, the smallest weighing only 1,000 g. We therefore saw no restrictions to start the thoracoscopic elongation in neonates as well. This series has demonstrated that neonates tolerate the procedure well. The smallest child weighed 1,600 g at the time of thoracoscopic elongation. Initially, we also started with giving the patients a gastrostomy. However, in many instances, the gastrostomy had to be taken down in order to facilitate a laparoscopic antireflux procedure 4–6 weeks later. As we started to perform the procedure in the first week of life, we decided to not place a gastrostomy any longer. In our first case, this ended with the stomach being pulled up into the thorax. In the past, it had always been the gastrostomy that had kept the stomach in place. In the following patient, an anterior gastropexy was performed laparoscopically with Vicryl 4 × 0 sutures. This efficiently kept the stomach down. However, on carrying out the laparoscopic antireflux procedure, we saw that the resorbable sutures in time had more or less been dissolved, leaving only fibrous bands between stomach and anterior abdominal wall. We thereafter changed to using Ethibond 4 × 0 non-resorbable sutures. So far this has efficiently kept the stomach down, even to such an extent that we had to release the gastropexy in our last patient in order to gain some more length to be able to make the anastomosis. Although this may seem contradictory, the benefits from making a watertight esophageal anastomosis outweigh the risk for a hiatal hernia that has to be corrected during the antireflux procedure.

The next issue to deal with after fulfilling the anastomosis is gastroesophageal reflux. Due to the traction, the gastroesophageal transition is stretched and pulled up into the thorax, undoing all antireflux properties. In spite of antireflux medication, and probably also due to marginal circulation, stenosis occurs, requiring dilation. Usually, the first dilation is planned for two weeks after the anastomosis, using a 8-mm dilation balloon, the second after 4 weeks using a 10 mm balloon, followed by an antireflux procedure. This may be challenging, because most of these patients have a micro-stomach, leaving little room for making a proper wrap. Important first step is to bring back the distal esophagus into the abdomen and narrowing the hiatal hernia. A one-step “mini” anterior wrap is created by approximating the anterior stomach wall against the esophagus at the level of the diaphragm and the diaphragm itself, instead of the usual two-step layer to create a sufficient length of intra-abdominal esophagus. Delaying the antireflux procedure for 4–6 weeks has two reasons: first, when the child is somewhat older, the tissues are less friable, and second, it will reduce the duration of the initial operation considerably.

Feeding in children with long gap esophageal atresia may be an issue. From one part, the small stomach only has a limited capacity which is not enough for adequate growth. Some of these children need to be on tube feeding for a prolonged period until the stomach has grown sufficiently and/or when solid feeds become possible. The children operated in the neonatal period could start drinking within 1–2 weeks after correction, although in some, the frequency remained on eight feeds for a longer time due to the small stomach. This early start reduces many of the feeding problems described after delayed start of feeding [3]. As soon as more solid food can be introduced, reflux, due to the limited capacity of the stomach, will be less obvious and sufficient energy intake becomes easier. Close collaboration with the dietician is important, and intake should be tailored to the individual patient.

More recently, there have been publications pointing out the negative side effects of anesthesia and surgery on neonates [12, 13]. This is also one of the reasons why antireflux surgery is delayed for 4–6 weeks. Currently, all patients are operated on under surveillance of NIRS and a-EEG to monitor cerebral oxygenation and brain activity. The outcomes look promising, but will be published in the near future.

In conclusion, management of long gap esophageal atresia seems to have taken a substantial step forward. Thoracoscopic treatment of long gap esophageal atresia not only is feasible, but also facilitates treatment in the neonatal period without the need for a gastrostomy and a hospitalization time approaching that of standard esophageal atresia and that seems more determined by prematurity and weight, than the surgical management itself.
